# Effect of Crude Venom of *Odonthobuthus doriae* Scorpion in Cell Culture using Ion Channel Modulators

**Published:** 2017

**Authors:** Sainaz Ghasemi, Amir Ahmad Salarian, Abbas Zare Mirakabadi, Somayeh Jafarinejad, Mahmoud Ghazi-Khansari

**Affiliations:** a *Department of Pharmacology, School of Medicine, Tehran University of Medical Sciences, Tehran, Iran.*; b *Department of Toxicology , AJA University of Medical Science, Tehran, Iran. *; c *Venomous Animals and Antivenom Production Department, Razi Vaccine and Serum Research Institute, Karaj, Iran.*

**Keywords:** Scorpion, *Odonthobuthus doriae*, 1321N1, Channel blocker, cell culture

## Abstract

Scorpion venom toxicity is one of the major medical concerns from old years, due to its influence on human activities and health. From many years ago a lot of researches established to examine different aspects of venom toxicity and its effects on different organs. During these years researchers are doing more specific studies on the cytotoxicity of scorpion venom. In Iran, *Odonthobuthus doriae,* the yellow scorpion is one of the major threats based on its neuro toxicity and severe pathophysiologic effects and researchers tried to find the mechanism of these neuro toxic effects. The previous studies have shown that in isolated organs the yellow scorpion venom is affecting the ion channels. Also some studies showed that this venom has severe cytotoxic effects on the cell lines with many ion channels like nerve cell lines.

In this study, the cytotoxic effect of the crude venom of *O.doriae* on the 1321N1 cell line (cancerous nerve cells) was studied. Primary cell cultured investigated in the presence of different ion channel blockers: Ouabain (1mmol as Na channel blocker), Nifedipin (100 µmol as Ca channel blocker), and TEA (40 mmol as K channel blocker) by MTT method. The result showed that the *O.doriae *crude venom has cytotoxic effect via Na channels.

## Introduction

During about 400 million years, Scorpions have successfully developed a large variety of bioactive peptides (1). Scorpions are widely spread around the world. There are about 1500 species of scorpions (2) from which approximately 25 species are dangerous to humans (3) especially for children and elderly (4), and envenomation by scorpion remains a serious health problem especially in tropical countries (5). The Iranian scorpion fauna consists of over 44 named species from 23 genera in two families of Buthidae and Scorpionidae. In Iran, the same as other parts of the world, there are a few known species of scorpions responsible for severe envenoming. Of these, at least seven species have been implicated in envenoming of human which are considered medically important (6). Among the most dangerous scorpions of Iran are those belonging to the family of Buthidae, such as *Odontobuthus *(7). Most of the scorpion toxins have been isolated from the venoms of scorpions in the family Buthidae. The genus *Odontobuthus* has three species: bidentatus, doriae, and odonturus. Specifically, *Odontobuthus doriae*, the yellow scorpion, can be found in the central and southern parts of Iran. Its sting can cause various effects ranging from local pain, inflammation and necrosis to muscle paralysis, which might be deadly for children (9, 10). Scorpion venoms are composed of a variety of biologically active components such as enzymes, peptides, nucleotides, lipids, mucoproteins, biogenic amines, and other unknown substances (11). The best studied group of scorpion venom components comprises the neurotoxins with polypeptides that recognize ion channels and receptors in excitable membranes (12). The biological effects of scorpion stings are mainly due to the presence of low-molecular-weight proteins in the venom that exert powerful effects on excitable cells (9). Although the main effects of scorpion venom are likely to be due to toxins that affect the opening of ion channels in nerve and muscles, the mechanism by which the venom from the Iranian yellow scorpion *O. doriae* causes its neuromuscular *in-vitro* effects, is not fully understood (9). Membrane channel blockers are known to control certain cellular behavior in the metastatic cascade (13) and also play a key role in cellular mitogenesis (14). This hypothesis may arouse the curiosity to study the antiproliferative and cytotoxic potentiality of scorpion venom (15). In Iran, *Odonthobuthus doriae*, the yellow scorpion is one of the major threats based on its neuro toxicity and severe pathophysiologic effects and researchers are trying to find the mechanism of these neuro toxic effects. The previous studies have shown that in isolated organs the yellow scorpion venom is affecting the ion channels. Also, some studies showed that this venom has severe cytotoxic effects on the cell lines with many ion channels like nerve cell lines. This study was carried out to investigate the role of ion channels in the cytotoxicity effect of this venom on nerve cells.

## Experimental

*Materials and Methods*

**Figure 1:**
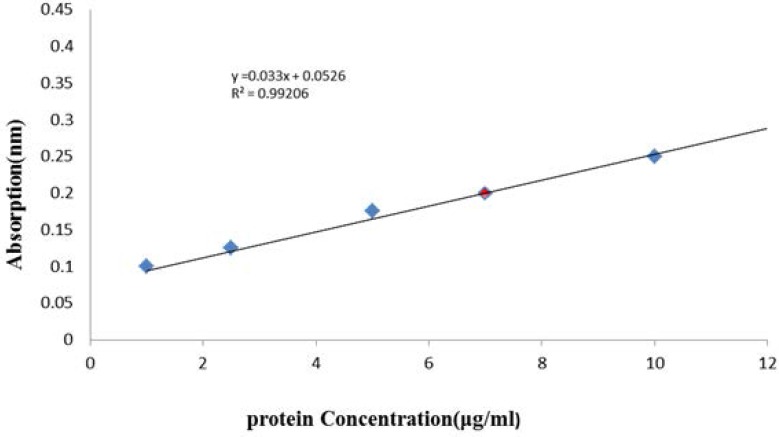
Calibration curve measured protein in the crude venom of *O. doriae *Bradford protein assay method

Venom preparation: the freeze dried venom was acquired from Razi institute, dissolved in distilled water, and placed in dialysis bag and also dialyzed against distilled water at 4 °C for 48 h. The venom solution was then centrifuged at 15000 rpm for 15 min and supernatant was collected for experiments. Protein content of supernatant was determined using Bradford protein assay method (1976), as modified by BioRad Inc., using bovine gamma globulin as a standard Figure (1). Enzyme specific activities and all other analyses were based on these protein concentrations. Standard curve was drawn using bovine serum albumin (BSA) for any new plate or assay.

1321N1 (Glial-like cell line derived from a human brain astrocytoma) cell line was acquired from Pasteur institute. 1321N1 cells were propagated in DMEM/F12 medium supplemented with 10% fetal bovine serum (FBS), and antibiotics penicillin (10,000 units/L) and streptomycin (100 mg/L). The cell line were incubated in flasks as monolayers and cultured at 37 °C with 5% CO2 in a fully humidified atmosphere. Three channel blockers: ouabain (1 mM as sodium channel blocker), tetraethyl ammonium (40 mM as potassium channel blocker, TEA), and verapamil (10 μmol/L as Calcium channel blocker) were used to block ion channels. Cytotoxicity assay for the purpose of examining the effects of scorpion venom on cell growth in the presence of ion channel blockers, using 3-(4,5-dimethylthiazol-2-yl)-2,5-diphenyl tetrazolium bromide (MTT) reagent, was performed. This assay is based on the cellular conversion of a tetrazolium salt (MTT) into a formazan product that is easily detected using a 96-well plate reader. Venom was initially diluted in PBS and then serial dilutions were prepared using cell free culture medium. Also, three ion channel blockers prepared using cell free culture medium. The cells (1.5 × 104 to 0.5 × 105 cells per well) were cultured in 96 wells plate and incubated for 24h at 37 ˚C with 5% CO2. After 24 h incubation, the cells were treated by three channel blockers and various concentrations of venom (0.5, 1, 1.5 μg/mL), and incubated for 24 h. After 24 h, cell culture medium in each well was replaced with 200 μL of medium containing 0.5 mg/mL MTT, followed by incubation at 37 °C for 3 h. DMSO was added into each well and the optical density of the base at 570 and that of the test at 650 nm were measured by a micro plate reader (Bio-Rad 550).The experiments were repeated three times. Cisplatin (50 μg/mL) was used as a cytotoxic agent for the control group. GraphPad Prism 3.02 version software was used for statistical analysis. One-way ANOVA and Newman-Keuls Multiple Comparison Test were used for data analyzing.

## Results and Discussion

**Figure 2:**
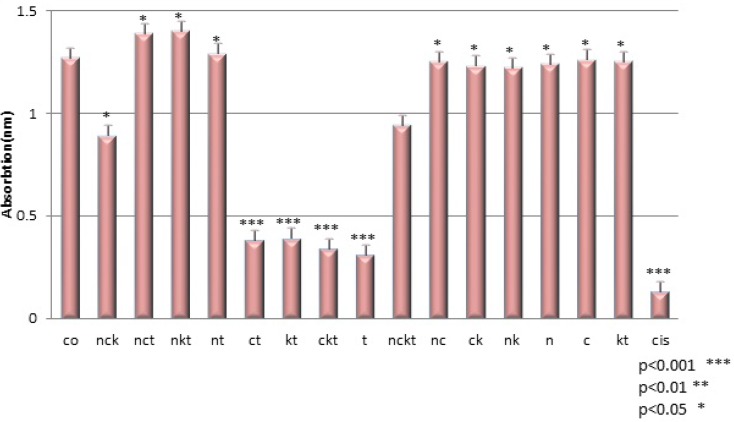
Cell viability after different treatments. Co(control), t(toxin:1µg/ml), n(Na^+^ channel blocker:1 mM Ouabain), c(Ca^2+^ channel blocker:10 µmol/L Verapamil), k(K^+^ channel blocker:40 mM TEA), cis(cisplatin:50 µ/mL

In this study, we investigated the cytotoxic effect of the crude Venom of *O. dories* scorpion on 1321N1 cultured cells, in the presence of different Ion channel blockers. The cytotoxic effect of venom was measured by cell count using MTT assay. At longer time points, 48 h, the cytotoxic effect was reduced, which might be due to venom denaturation caused by rising temperature in aqueous solutions. That is why a 24 h exposure was used to determine the cytotoxicity of venom in the most of the experiments. As it has been shown in Figure 2, cell survivals measured in the presence of three channel blockers and venom are compared with controls. Two control groups were chosen in this experiment: Group 1 that just contains cells, Group 2 that contains cells treated by venom. As shown in Figure 2, cell survival changed after cells were treated for 24 h by Ca^2+^ and K^+^ channel blockers (P < 0.001). But, when cells were treated by Na^+^ and/or K^+ ^channel blockers, and Ca and/or Na^+^ channel blockers, there was no change in cell survival (P>0.05)( Figure 2). But after 24h treatment of cells with Ca^2+^ and K^+^ channel blockers, the viability of cells decreased, compared to control group (P < 0.001 ), which shows Na channels are responsible for the cytotoxic effects of the crude venom of *O. doriae* on 1321N1 cell line. Previous studies showed some evidence that there is relation between cytotoxic effects of *O. doriae* venom and ion channels (8, 3).

The next studies will be focused on finding the active fraction(s) of the crude venom, responsible for cytotoxic effects in cultured cells, *via* Na^+^ channels.
